# Cost‐Effectiveness of Family Conferences to Reduce Polypharmacy in Frail Older Adults

**DOI:** 10.1111/jgs.19606

**Published:** 2025-06-27

**Authors:** Joseph Montalbo, Charalabos‐Markos Dintsios, Jens Abraham, Eva Drewelow, Manuela Ritzke, Achim Mortsiefer, Birgitt Wiese, Petra Thürmann, Stefan Wilm, Andrea Icks, COFRAIL study group

**Affiliations:** ^1^ Medical Faculty, Institute for Health Services Research and Health Economics Centre for Health and Society, Heinrich‐Heine‐University Düsseldorf Düsseldorf Germany; ^2^ Medical Faculty, Institute for Health and Nursing Science Martin Luther University Halle‐Wittenberg Halle Germany; ^3^ Institute of General Practice University Medical Center Rostock Rostock Germany; ^4^ Faculty of Health, Department of Medicine Institute of General Practice and Primary Care, Witten/Herdecke University Witten Germany; ^5^ WG Medical Statistics and IT‐Infrastructure Institute of General Practice, Hannover Medical School Hannover Germany; ^6^ Department of Clinical Pharmacology School of Medicine, Faculty of Health, Witten/Herdecke University Wuppertal Germany; ^7^ Medical Faculty Institute of General Practice, Heinrich‐Heine‐University Düsseldorf Düsseldorf Germany

**Keywords:** COFRAIL, cost‐utility, deprescribing, health economic evaluation, shared decision‐making

## Abstract

**Background:**

Cost‐effectiveness of family conferences on deprescribing with joint prioritization of treatment goals in primary care has not been investigated so far. We assessed cost‐effectiveness in the cluster‐randomized controlled COFRAIL trial conducted with general practitioners and 521 older frail patients with polypharmacy cared for at home in Germany.

**Methods:**

Hospital admissions averted and quality‐adjusted life years (QALYs) gained were associated with costs from the German Social Insurance perspective. We applied adjusted GLM regressions with specified distributions to estimate group differences on imputed data, plotted bootstrap cost‐outcome pairs by simulated resampling of the study population to illustrate uncertainty and calculate the probability of cost‐effectiveness given a willingness‐to‐pay threshold, and assessed robustness in sensitivity analyses.

**Results:**

Intervention‐related costs were €391 (US$459) per capita. On 100 people, the COFRAIL intervention had about 7 more hospital admissions (95% CI: −12; 26), 2 QALYs gained (95% CI: −1; 6), and additional costs of €117,681 (95% CI: −28,838; 264,201)/US$138,027 (95% CI: −33,824; 309,880) or €124,866 (95% CI: −12,649; 262,380)/US$146,455 (95% CI: −14,836; 307,745) without or with hospital costs, respectively, compared to usual care. By bootstrapping, we observed the COFRAIL intervention to have higher costs and more hospital admissions with a relative frequency of 28%–78%, or in terms of QALYs 57%–91%. The COFRAIL intervention had additional costs of €50,966 (US$59.778) per QALY gained with a 46% probability of being cost‐effective at a willingness to pay of €45,000/QALY (≈US$50,000/QALY).

**Conclusion:**

The COFRAIL intervention affected QALYs rather than hospital admissions after 12 months. The intervention tended to be associated with higher costs and QALYs but was less likely to be cost‐effective than usual care at commonly used willingness‐to‐pay thresholds. Long‐term cost‐effectiveness should be assessed.

**Summary**

- Key points
  ○ Frail older adults with polypharmacy are at high risk for adverse health outcomes, and deprescribing is considered an option to reduce this risk.
  ○ We found that family conferences on deprescribing to reduce polypharmacy in frail older adults cared for at home in Germany was less likely cost‐effective at commonly used willingness‐to‐pay thresholds regarding health‐related quality of life (QALYs) than usual care.
  ○ Cost‐effectiveness analyses of interventions to reduce polypharmacy in high‐risk populations for adverse health outcomes can put the contribution of these interventions into perspective.
- Why does this paper matter?
  ○ The present health economic evaluation assessed the cost‐effectiveness of family conferences on deprescribing with joint prioritization of treatment goals within a cluster‐randomized controlled trial versus usual care at the 12‐month follow‐up. The COFRAIL intervention was delivered by general practitioners for frail older care‐dependent patients with polypharmacy living at home.
  ○ We performed both a cost‐effectiveness analysis and a cost‐utility analysis to understand the reach of the COFRAIL intervention on patient‐relevant outcomes (hospital admissions averted, quality‐adjusted life years gained) and on costs from the perspective of the German Social Insurance (payers in German health care system).
  ○ We addressed challenges in our dataset by employing multiple imputation techniques to replace missing data, applying adjusted GLM regressions with specified distribution to account for non‐normally distributed data, using bootstrap procedures to assess uncertainty, and performing sensitivity analyses to test the robustness of our results.
  ○ After 12 months of observation, we found the COFRAIL intervention more likely to affect QALYs than hospital admissions. It tended to be more costly (due to higher healthcare costs rather than the costs of the intervention itself) but also more effective in terms of QALYs than usual care. Cost‐effectiveness was less likely observed at commonly used willingness‐to‐pay thresholds per QALY gained. Our results open the way to model long‐term cost‐effectiveness to support decision‐makers in their comparison of the COFRAIL intervention with usual care.
  ○ Cost‐effectiveness analyses of interventions to reduce polypharmacy in high‐risk populations for adverse health outcomes can put the contribution of these interventions into perspective.
  ○ Furthermore, since transferability of these results is given for modern health care systems, the conclusions drawn by the health economic evaluation can be relevant for other western countries and the USA as well.

## 1 Introduction

Adults aged 65 years and older with a vulnerable health status classified as frailty [1] and with polypharmacy (defined as medication intake of at least five drugs per day [2]) are at high risk for adverse health outcomes including falls, hospitalizations, and death [1, 3].

Polypharmacy is one risk factor besides higher age and frailty contributing to adverse drug reactions leading to hospital admissions [4]; therefore, the benefit/risk ratio of each drug prescribed to a frail older adult has to be carefully assessed. One intervention approach that has been effective in improving the appropriate use of polypharmacy in older adults is to reduce the number of inappropriate drugs (i.e., deprescribing) based on medication reviews [5].

In the literature on health economic evaluations, intervention programs focusing on deprescribing under consideration of patients' preferences were observed to have a positive impact on patient‐relevant outcomes such as quality‐adjusted life years (QALYs) and also on costs. The interventions were described to promote shared decision‐making, to be patient‐centered, or to involve a collaboration of patients with general practitioners led by pharmacists [6, 7, 8, 9]. Deprescribing of sedatives [6] and nonsteroidal anti‐inflammatory drugs [7] improved QALYs and decreased costs versus the comparator (i.e., deprescribing was dominating the comparator) in community‐dwelling older adults after 12 months, as shown in recent simulation models in Canada. Even after 6 months, similar results were found through medication reviews in older adults with polypharmacy in a trial‐based analysis in Spain [8]. However, this was inconsistent with another 6‐month trial‐based analysis including an older population with polypharmacy in the Netherlands, as a loss of QALYs was offset against cost savings through the intervention [9]. Evidence suggested that the intervention was either dominating the comparator [6, 7, 8] or was cost‐effective at reported thresholds in terms of what a payer might be prepared to save for a QALY loss [9]. While evidence exists that deprescribing is favored over the comparator in terms of QALYs and costs in community‐dwelling older adults with or without polypharmacy, little is known in a population composed of older adults in need of care at home with a poorer health state, that is, patients with polypharmacy as well as with frailty.

In the COFRAIL study, this population group was less receptible to deprescribing compared to usual care when measuring patient safety in terms of reduction of hospital admissions after 12 months of observation [10]. However, expected improvements in QALYs are considered to be reasonable, as deprescribing in the COFRAIL study was embedded in a family conference, that is, a shared decision‐making process involving general practitioners, patients, and family caregivers, where patient needs were prioritized [11]. This required a training of general practitioners on the appropriate use of a deprescribing manual [12] and a geriatric toolbox, which was developed for needs assessment of problem areas of older patients with regard to physical and mental limitations (i.e., performance in everyday life, social environment and emergency plan, mobility and agility, falls, vertigo, vision and listening restriction, urinary and fecal incontinence, etc.) and nonpharmacological options (e. g., physiotherapy, occupational therapy, medical foot care, hearing aids, visual aids, walking aids, nursing service, and preventive check‐ups) focusing more on the everyday limitations of diagnoses (e.g., falls) and their prevention rather than on indications itself (e.g., cardio‐vascular problems) [11]. In return, the benefits in QALYs may reduce healthcare costs and offset the costs related to the COFRAIL intervention (as it was labor‐intense in terms of training and delivery [11]).

To investigate how costs and patient‐relevant outcomes were associated with frailty and polypharmacy in older adults living at home, we performed a health economic evaluation alongside the COFRAIL study. The aim was to assess the cost‐effectiveness (i.e., the comparison of the relative costs and effects of different interventions) of the COFRAIL intervention versus usual care at the 12‐month follow‐up considering two key outcomes: QALYs and patient safety in terms of reduction of hospital admission.

## 2 Methods

### 2.1 The COFRAIL Study

A cluster‐randomized controlled trial was conducted to assess the effectiveness of deprescribing within family conferences (COFRAIL intervention) primarily in terms of hospital admissions versus usual care in frail older patients with polypharmacy living at home [13]. The COFRAIL study was initiated in 2018 and included a 12‐month follow‐up of the participating patients (2019: first patient in; 2021: last patient out) [10].

Patients were recruited by general practitioners from two areas of Germany (around Düsseldorf in the west and Rostock in the northeast) if they met the following inclusion criteria: (i) screened positive for frailty, that is, had level 5 to 7 on the Canadian Study of Health and Aging Clinical Frailty Scale [14], (ii) at least 70 years of age, (iii) regular intake of at least five different pharmaceuticals, (iv) need of care (i.e., existing level of care dependency between 1 and 5 according to the medical service of the German social care insurance or a comparable status assessed by general practitioners), and (v) received care at home by family members and/or ambulatory care service.

Randomization took place on the level of general practitioner practice (cluster), that is, all patients recruited from the same general practitioner practice were randomly assigned to the same group, either the intervention group or the control group. Out of 623 recruited patients from 114 practices who entered randomization, 521 patients from 110 practices were assessed at baseline. The remaining difference was the number of dropouts prior to baseline assessment. Patients in the intervention group (*n* = 272, from 56 practices) received the COFRAIL intervention whereas patients in the control group (*n* = 249, from 54 practices) received usual care.

The COFRAIL intervention included a training program (two mandatory sessions, one voluntary session) for general practitioners by qualified personnel (i.e., general practitioners, scientific staff, experts for care needs) on how to deprescribe pharmaceuticals using a deprescribing manual [12] and how to use a geriatric toolbox of non‐pharmacological interventions (e.g., fall prevention) tailored to patient needs within the concept of a family conference, that is, a shared decision‐making process of general practitioners, patients, and family caregivers [11]. Patients in attendance with caregivers and/or the ambulatory care service received up to three family conferences implemented by general practitioners within 9 months, lasting up to 45 min each. The first family conference was used to conduct a medication review and to deprescribe drugs in mutual agreement if indicated by the deprescribing manual, the second to discuss and implement non‐pharmacological interventions from the geriatric toolbox, and the third to individually internalize the contents of the previous ones [11]. General practitioners received support by a medical assistant during intervention delivery if needed. Usual care comprised only voluntary training for general practitioners on geriatric topics that did not interfere with the COFRAIL intervention.

The COFRAIL study was registered on International Clinical Trials Registry Platform (DRKS00015055) and was approved by the ethics committees of the study centers Rostock and Düsseldorf.

### 2.2 Design of the Health Economic Evaluation

The health economic evaluation was conducted alongside the COFRAIL study assessing the cost‐effectiveness of the COFRAIL intervention versus usual care at the 12‐month follow‐up. A brief description of our approach can be found in the study protocol of the trial [13]. This research adhered to the Consolidated Health Economic Evaluation Reported Standards Statement (CHEERS), an internationally established guideline to ensure health economic evaluations are identifiable, interpretable, and useful for decision making [15].

The base case included all 521 patients, who have been randomized and examined at baseline (intention‐to‐treat‐population; intervention group: 272, control group: 249). In a different scenario, patients were considered only if they did not dropout from the study, that is, if they were surveyed at least at baseline and 12 months after baseline. In addition, patients in the intervention group were not considered as dropouts if they received at least two family conferences (per‐protocol‐population: 385; intervention group: 200, control group: 185).

Cost‐effectiveness was assessed in terms of additional costs per hospital admission averted as outcome in a cost‐effectiveness analysis and in terms of additional costs per QALY gained as outcome in a cost‐utility analysis, a well‐established subtype of a cost‐effectiveness analysis.

The analyses were carried out from the perspective of the German Social Insurance as we considered costs related to the COFRAIL intervention and healthcare utilization from the statutory health insurance and statutory long‐term care insurance.

We did not discount costs and outcomes since the COFRAIL intervention lasted only a year [16]. Data collection was done by study nurses via home visits (or telephone surveys during the COVID‐19 pandemic) at baseline as well as 6 and 12 months after baseline.

### 2.3 Outcomes

#### 2.3.1 Hospital Admissions

The outcome parameter of the cost‐effectiveness analysis was the primary endpoint of the COFRAIL trial as a measure for patient safety at the 12‐month follow‐up [10]. Although the COFRAIL intervention was not associated with an improvement in patient safety compared to usual care, we included this outcome to investigate the cost‐effectiveness of the COFRAIL intervention in case of cost savings. We calculated the outcome as the sum of hospital admissions measured 6 and 12 months after baseline (each point in time with a 6‐month recall period). Hospital admissions were assessed in two ways: (I) by patients' self‐report, based on a modified version of a validated questionnaire, and (II) by documentation of general practitioners (i.e., questionnaire and drop‐out form). In our base case, we used self‐reported data to avoid contradictory information or the risk of double counting due to hospital admissions being recalled by patients and general practitioners at different times of data collection. In a different scenario, we used the general practitioner questionnaire as the data source for the outcome to account for the potential recall bias in self‐assessed questionnaires.

#### 2.3.2 Quality‐Adjusted Life Years

The outcome parameter of the cost‐utility analysis was calculated from the self‐assessed health status via the preference‐based EQ‐5D‐5L questionnaire [17] measured at baseline as well as 6 and 12 months after baseline. The health status was converted to a utility weight by using the actual German tariff [18]. We rescaled negative utility weights to range between 0 and 1 [19], with the former as the smallest and the latter as the highest utility weight in the data corresponding to death and full health, respectively. In a different scenario, we used values of the EQ‐visual analogue scale to directly measure the health status and to avoid rescaling. We divided the values by 100 to transform scores ranging from 0 to 1, in which 0 indicated the worst and 1 the best possible health status.

To determine total QALYs in the base case at the 12‐month follow‐up, we applied linear interpolation between utility weights to calculate the area under the curve [20]. Thus, QALYs capture differences in quality of life over 12 months.

### 2.4 Costs

Costs were calculated in Euros (€) for the reference year 2019 by multiplying quantities and prices from published sources and official German statistics (€ amounts were converted into 2019 US dollars at purchasing power parity unless otherwise specified). We provide a list of 2019 prices (shown in Table S1) and describe our approach to quantifying and evaluating healthcare resource use (shown in Table S2).

#### 2.4.1 Intervention‐Related Costs

The COFRAIL intervention costs were composed of a fixed and a variable component. We calculated the fixed component based on the following information from study centers: costs of training (i.e., room and catering), cost of manuals/information brochures (i.e., materials, printing, and postage), time expenditure of personnel (general practitioners, scientific staff, and staff from care centers) to organize and perform the training, personnel expenses due to traveling to/from training, and time expenditure of general practitioners participating in the training. We calculated average costs per patient in the intervention group based on the intention‐to‐treat population. The variable component was the average cost per family conference calculated as the ratio of total time expenditure to deliver family conferences to patients (including preparation and follow‐up) and the total number of family conferences delivered. Taking into account the results of the pilot study conducted within the intervention development, a family conference would generate time expenses of 1.25–1.75 h for general practitioners in addition to a time expenditure of 1 h for medical assistants [11]. Time expenditure was evaluated based on gross hourly wages plus social contributions of the employer [21] as we expected these expenses to arise in association with the employment of the workforce.

#### 2.4.2 Healthcare Costs

The above‐mentioned questionnaire [22, 23] was applied in the COFRAIL study to quantify healthcare resource use due to outpatient medical care, therapeutic treatments, admissions to hospital ambulances (emergency), rehabilitation, support in households, pharmaceutical intake, medical technical aids, and services of the long‐term care insurance. Hospital admissions were considered as healthcare resource use only in the cost‐utility analysis as they were considered as outcomes in the cost‐effectiveness analysis (to avoid double counting). Healthcare resource use was measured at baseline as well as 6 and 12 months after baseline with a 6‐month recall period each point in time except for therapeutic treatments and support in households (4 weeks), and pharmaceutical intake (7 days).

#### 2.4.3 Aggregated Annual Costs

To calculate aggregated annual costs at the 12‐month follow‐up, we summed up healthcare costs across time (i.e., 6 and 12 months after baseline) and categories. In case of healthcare costs with shorter recall periods than 6 months (i.e., therapeutic treatments and support in households), we applied linear extrapolation except for pharmaceutical intake as it depended on its duration. In addition to healthcare costs, intervention costs were considered to calculate aggregated annual costs at the 12‐month follow‐up for the intervention group.

### 2.5 Additional Variables

To describe population characteristics at baseline, additional data were included in the analyses despite outcomes and costs: namely sex, age, housing situation, educational status, health insurance status, BMI, existing disability, existing diseases as an indicator for 12 diseases at most (median value), existing level of care dependency, and recruitment site. We collected the information primarily using a self‐assessment questionnaire. In the case of missing BMI, we completed the information from the documentation of the general practitioner. To account for the general practitioner practice (cluster) in the analyses, we collected information on its medical specialty (i.e., general medicine, internal medicine, general and internal medicine, other, or unknown) in addition to the identification number.

### 2.6 Missing Data

Multiple imputation was used to replace missing data in costs, utility weights, hospital admissions (at baseline as well as 6 and 12 months after baseline), and additional variables (at baseline) assuming that the cause of the data unavailability could be explained by variables with complete information: i.e., that the data were missing at random [24]. We followed the rule of thumb recommending the number of complete versions of the dataset generated with different imputed values (imputed dataset) to be at least equal to the percentage of missing observations [25]. Further information about our imputation model is provided elsewhere (shown in Table S3). For those who died during the study, utility weights corresponding to death were used [26] and no hospital admissions or healthcare costs were calculated after the fatal event. Further steps to prepare the data prior to multiple imputation are explained elsewhere (shown in Table S4). In different scenarios, we run analysis without patient deaths (per‐protocol population) and without using multiple imputation.

### 2.7 Estimating Mean Incremental Outcomes and Costs

Mean incremental outcomes and costs per capita were calculated as the difference in mean outcomes and costs between the intervention and control group. We achieved this by computing the margins from a Generalized Linear Model (GLM). We applied a GLM with gamma distribution and log link function to estimate mean incremental costs [27] and mean incremental QALYs (outcome of the cost‐utility analysis), whereas we used a GLM with Poisson distribution and log link function to estimate mean incremental hospital admissions (outcome of the cost‐effectiveness analysis). The link was selected after performing statistically non‐significant (*p* > 0.05) Pregibon link test [28] next to (modified) Hosmer and Lemeshow test and Pearson correlation, respectively.

We adjusted for baseline differences by performing GLM regressions with control variables including the baseline value of additional variables (see paragraph “*Additional variables*”) and the baseline value of either costs or outcomes (i.e., hospital admissions in the cost‐effectiveness analysis, utility weights in the cost‐utility analysis). Adjusting for baseline differences seemed to be a reasonable approach [29], especially because patients in the COFRAIL study were randomized on cluster level. The identification number of the general practitioner practice was used to take into account the cluster (clustered standard errors) when calculating 95% confidence intervals. In a different scenario, we exclude control variables from GLM regressions.

Mean incremental costs and mean incremental outcomes as well as confidence intervals resulting from the GLM regressions were averaged across all imputed datasets. The results were presented on 100 patients to simplify the interpretation.

Positive mean incremental outcomes of the cost‐utility analysis (QALYs) were interpreted as benefits of the COFRAIL intervention compared to usual care, whereas positive mean incremental outcomes of the cost‐effectiveness analysis (number of hospital admissions) were not in favor of the COFRAIL intervention. In order to change the interpretation of the latter in a more comprehensible manner, we presented the number of hospital admissions averted such that higher incremental values would correspond to an improvement in patient safety.

### 2.8 Estimating Incremental Cost‐Effectiveness Ratio and Incremental Cost‐Utility Ratio

The incremental cost‐effectiveness ratio and incremental cost‐utility ratio were calculated by dividing mean incremental costs by mean incremental outcomes. Their estimate would suggest how many additional resources in terms of costs would be required to achieve one unit of outcome with the COFRAIL intervention compared to usual care.

### 2.9 Uncertainty and Probability of Cost‐Effectiveness

Uncertainty in the cost‐effectiveness of the COFRAIL intervention was assessed by running a bootstrap procedure (i.e., a specific resampling technique). From each imputed dataset, 1000 bootstrap samples were drawn [30], separately according to group assignment with the same random seed (i.e., 12,345). Mean incremental costs and mean incremental outcomes were calculated in each bootstrap sample and then averaged across all imputed datasets, yielding a dataset of 1000 estimates varying from the original result. The mean incremental cost‐outcome pairs were plotted on cost‐effectiveness planes and cost‐utility planes [31]. We calculated relative frequencies in each quadrant of the cost‐effectiveness/cost‐utility planes to determine the tendency of the COFRAIL intervention compared to usual care in terms of costs and outcomes.

The probability of the COFRAIL intervention being cost‐effective compared to usual care was calculated as the share of incremental cost‐effectiveness ratio and incremental cost‐utility ratio estimates below a willingness‐to‐pay threshold, which was illustrated on a cost‐effectiveness acceptability curve [32]. Since willingness‐to‐pay thresholds have never been formally adopted in Germany, we investigated the probability of cost‐effectiveness at €100, €1000, €10,000, and €100,000 (US$117.30, US$1173, US$11,730, and US$117,300) per hospital admission averted. In terms of willingness‐to‐pay per QALY gained, we referred to thresholds commonly used in the UK [33], that is, ≈€24,000 and ≈€35,000 (GB£20,000 and GB£30,000; exchange rate GB£1 = €1.18190, 31th December 2019 [34]) and in the US [35], that is, ≈€45,000 (US$50,000; exchange rate US$1 = €0.89127, 31th December 2019 [34]).

### 2.10 Sensitivity Analysis

Here, we deviated from the base case approach to investigate the impacts of specific changes on the robustness of our base case results. In sensitivity analyses (SA)‐I, a GLM model was applied without adjusting for baseline differences (but with clustered standard errors). In SA‐II, all patients dropping out from the study (including patient death) were removed from the analysis, that is, only patients in the per‐protocol population remained. In SA‐III, only patients of the per‐protocol population with complete data prior to multiple imputation were eligible for the analysis (intervention group: 150, control group: 138).

In additional analyses, we used general practitioner questionnaires to calculate hospital admissions in the cost‐effectiveness analysis (SA‐IV) and values of the EQ‐visual analogue scale to calculate QALYs in the cost‐utility analysis (SA‐V). The imputation model was adapted for SA‐II, SA‐IV, and SA‐V. We reported all changes from the base case approach regarding the imputation model in Table S5.

We used Stata (Version 15.1, StataCorp LLC, College Station, TX, USA) to prepare the data and perform all subsequent analyses.

## 3 Results

### 3.1 Descriptive Analysis

Based on the percentage of missing observations in the data varying between 0.38% to 24%, we generated 25 imputed datasets. The characteristics of the population by group at baseline (with respect to outcomes, costs, and additional variables) and at the 12‐month follow‐up (with respect to outcomes and costs) are shown in Table 1.

**TABLE 1:** Baseline characteristics and outcomes/costs after 12 months.

	Intervention group (*n* = 272)	Control group (*n* = 249)
Baseline		
Females (%)	67 [61; 72]	70 [65; 76]
Age (years)	84 [83; 84]	83 [83; 84]
Living alone (%)	48 [42; 54]	45 [38; 51]
Low education level (%)	70 [64; 75]	69 [64; 75]
Insured in the SHI (%)	95 [92; 97]	93 [90; 97]
BMI (kg/m)	28]28; 29]	29 [28; 30]
Existing disability (%)	53 [47; 59]	55 [48; 61]
Existing diseases^a^ (%)	60 [55; 66]	55 [48; 61]
Care dependency^b^ (%)	82 [77; 86]	83 [78; 88]
Recruited around Duesseldorf (%)	66 [61; 72]	62 [56; 68]
Medical specialty^c^ (%)		
General medicine	61	55
Internal medicine	28	37
General and Internal medicine	5	3
Other or unknown	6	5
Hospital stays (%)	39 [29; 48]	41 [32; 50]
Utility weights^d^	0.86 [0.84; 0.87]	0.85 [0.83; 0.87]
Semiannual costs in € (excl. hospital)	5467 [4736; 6197]	4930 [4553; 5308]
Semiannual costs in US$ (excl. hospital)	6412 [5554; 7269]	5782 [5340; 6226]
Semiannual costs in € (incl. hospital)	6940 [6106; 7773]	6496 [5923; 7070]
Semiannual costs in US$ (incl. hospital)	8140 [7162; 9117]	7619 [6947; 8293]
12 months after baseline		
Hospital stays (%)	66 [49; 83]	61 [46; 75]
QALYs	0.82 [0.79; 0.84]	0.79 [0.77; 0.82]
Annual costs in € (excl. hospital)	9467 [8416; 10,518]	8521 [7674; 9368]
Annual costs in US$ (excl. hospital)	11,104 [9871; 12,337]	9994 [9001; 10,988]
Annual costs in € (incl. hospital)	11,855 [10,563; 13,147]	10,722 [9704; 11,740]
Annual costs in US$ (excl. hospital)	13,905 [12,389; 15,420]	12,576 [11,382; 13,770]

*Note*: Data were mean, 95% CIs in brackets, numbers were rounded.

Abbreviations: BMI, body mass index; GP, general practitioner; SHI, statutory Health Insurance.

^a^ Indicates whether patients had up to 12 diseases (median value).

^b^ Indicates existing level of care dependency between 1 and 5.

^c^ Indicates the medical specialty of the GP practice.

^d^ Calculated from the health status of the EQ‐5D‐5L questionnaire.

Participants were mostly female (about 70%) and had a mean age of approximately 80 years. On average, annual aggregated costs including hospital costs amounted to €11,855 per capita (95% CI: 10,563 to 13,147)/US$13,905 (95% CI: 12,389–15,420) in the intervention group (healthcare costs = €11,464 (US$13,446); intervention costs = €391 (US$459)) and €10,722 per capita (95% CI: 9704 to 11,740)/US$12,576 (95% CI: 11.382–13.770) in the control group at the 12‐month follow‐up. This shows that costs related to the intervention only had a very small share (about 3%) relative to healthcare costs (about 97%) in the intervention group. From the mean intervention costs of €391 (US$459) per capita, about €118 (US$138) and €110 (US$129) correspond to the fixed component and the variable component, respectively.

Healthcare costs were distributed similarly between groups (shown in Figure S1). We observed the costs of long‐term care insurance to have the highest share of healthcare costs, followed by costs due to hospital admission and pharmaceutical intake.

### 3.2 Cost‐Effectiveness Analysis Base Case

The results of the cost‐effectiveness analysis indicated that the COFRAIL intervention did not, on average, improve patient safety in terms of averted hospital admissions per capita and was associated with higher costs per capita than usual care at the 12‐month follow‐up. On 100 patients, this would translate into about 7 more hospital admissions (95% CI: 12 less to 26 more) and additional costs of €117,681 (95% CI: −28,838 to 264,201)/US$138,027 (95% CI: −33,824; 309,880) at the expense of the intervention group, as shown in Table 2. The incremental cost‐effectiveness ratio was not calculated because we observed the COFRAIL intervention to be dominated by usual care.

**TABLE 2:** Base case results on 100 patients.^a^

CEA			CUA		
Incremental cost	Incremental outcome	ICER	Incremental cost	Incremental outcome	ICUR
Currency	Hospital admissions averted	€ or US$/hospital admissions averted	Currency	QALYs	€ or US$/QALY
€117,681 [−28,838; 264,201]	−7.17 [−26.40; 12.05]	Dominated	€124,866 [−12,649; 262,380]	2.45 [−1.13; 6.03]	€50,965.71/QALY
US$138,027 [−33,824; 309,880]			US$146,455 [−14,836; 307,745]		US$59,777.66/QALY

Abbreviations: CEA, cost‐effectiveness analysis; CUA, cost‐utility analysis; ICER, incremental cost‐effectiveness ratio; ICUR, incremental cost‐utility ratio.

^a^ Regression was performed with control variables. 95% CIs were obtained from the GLM.

**FIGURE 1:**
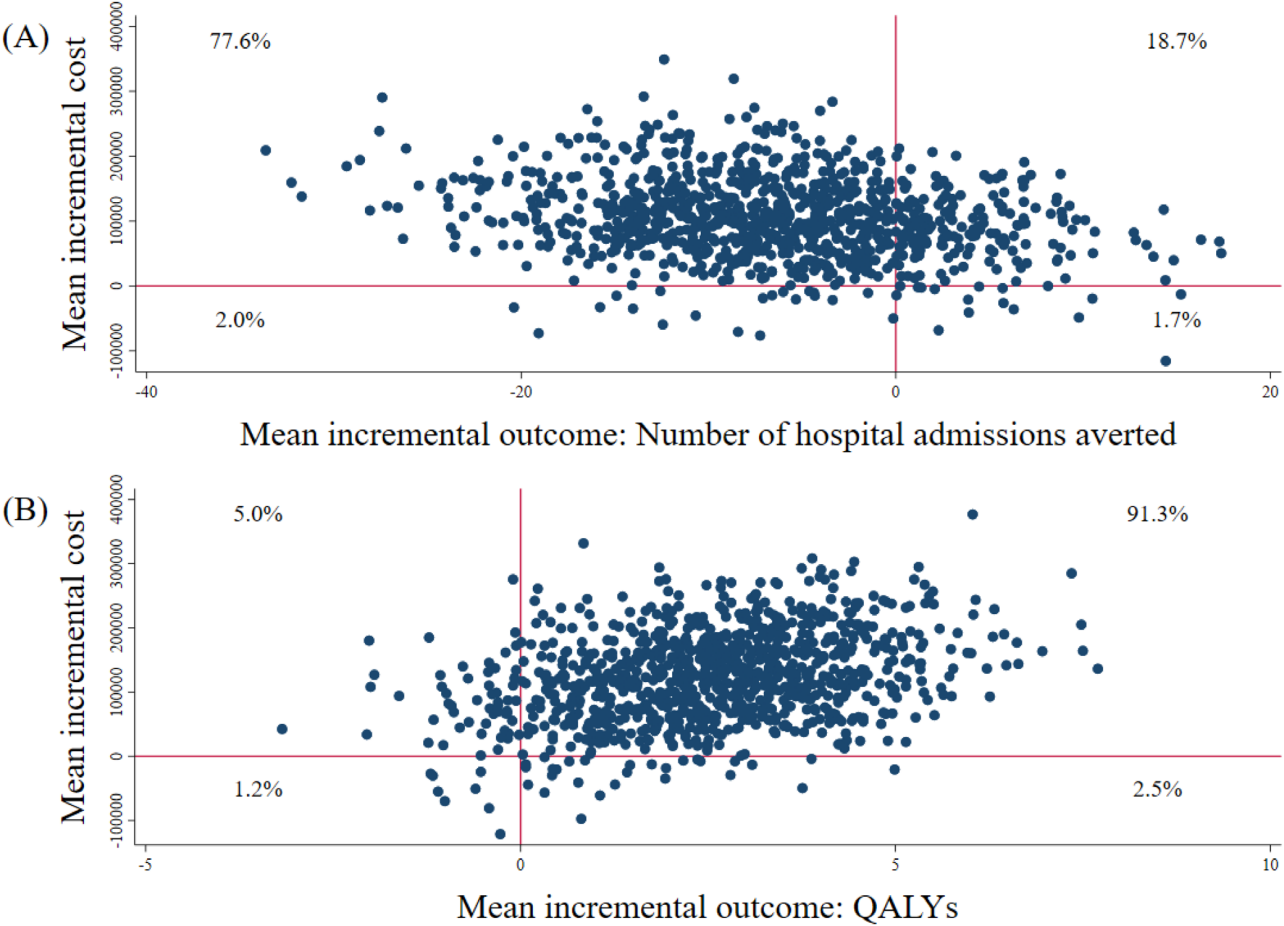
Cost‐effectiveness plane (A) and Cost‐utility plane (B) on 100 patients in the base case. CE plane, cost‐effectiveness plane; CU plane, cost‐utility plane; QALYs, quality‐adjusted life years. The figure shows the simulated distribution of cost‐effectiveness (A) and cost‐utility (B) pairs by bootstrapping subdividing the plane in four quadrants depending on costs (higher or lower than the comparator) and effects (better or worse than the comparator). Points of the scatter plot in the North‐East quadrant are considered to be cost‐effective (intervention with better effects and higher costs than the comparator). In analogy, points in the South‐East quadrant reflect dominance of the intervention over the comparator (better effects and lower costs), points in the North‐West quadrant demonstrate the opposite (intervention with worse effects and higher costs than the comparator and thereby dominated by the comparator), and finally points in the South‐West quadrant show less costs with worse effects of the intervention against the comparator.

This was highlighted in the cost‐effectiveness plane in Figure 1A, as the vast majority of mean incremental cost‐outcome pairs were plotted on the upper left quadrant of the cost‐effectiveness plane (relative frequency at 78%), suggesting that the COFRAIL intervention had a tendency to be less effective but more costly than usual care.

### 3.3 Cost‐Utility Analysis Base Case

The results of the cost‐utility analysis suggested that the COFRAIL intervention was on average associated with marginally higher QALYs per capita and higher costs per capita than usual care at the 12‐month follow‐up. On 100 patients, this would translate into about 2 QALYs gained (95% CI: −1 to 6) and additional costs of €124,866 (95% CI: −12,649 to 262,380)/US$146,455 (95% CI: −14,836; 307,745) for the intervention group (shown in Table 2). The incremental cost‐utility ratio suggested that the COFRAIL intervention would have additional costs of €50,966 (US$59,778) per QALY gained compared to usual care.

**FIGURE 2:**
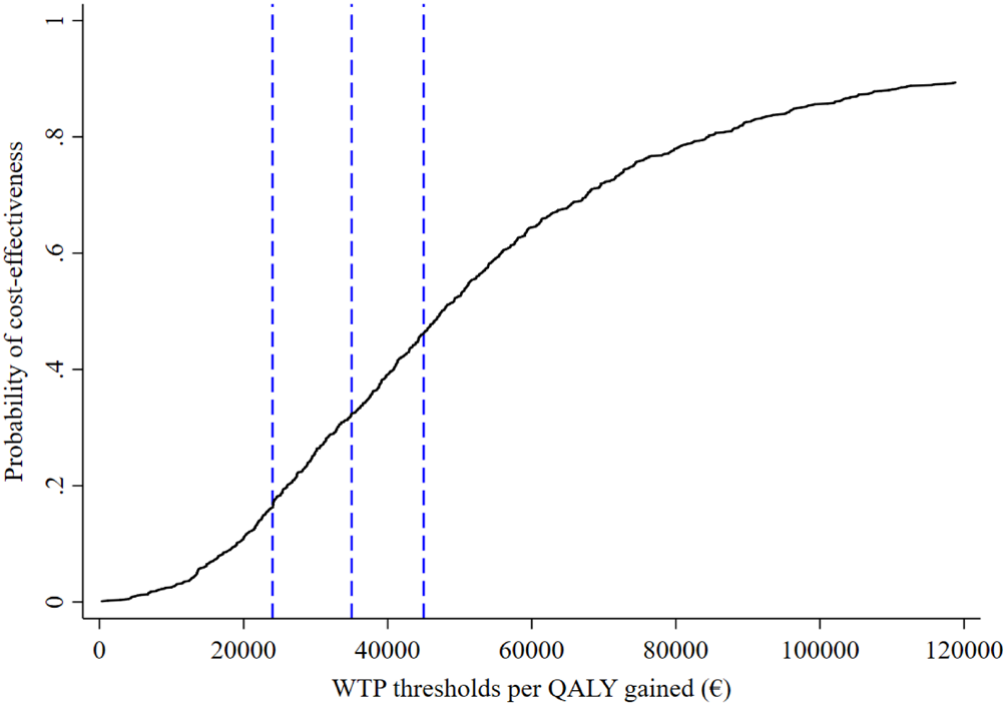
Cost‐effectiveness acceptability curve. QALYs, quality‐adjusted life years; WTP, willingness‐to‐pay. The figure depicts the probability of the intervention being cost‐effective against a range of willingness‐to‐pay with the dotted lines representing different internationally established thresholds for the willingness‐to‐pay.

In the cost‐utility plane (shown in Figure 1B), the vast majority of mean incremental cost‐outcome pairs were plotted on the upper right quadrant of the cost‐utility plane (relative frequency at 91%), that is, the COFRAIL intervention had a tendency to be more effective but also more costly than usual care. Assuming a willingness‐to‐pay threshold of €24,000 (≈US$27,000) and €35,000 (≈US$40,000) per QALY gained, the probability of the COFRAIL intervention being cost‐effective compared to usual care would be below 35%, as illustrated on the cost‐effectiveness acceptability curve (Figure 2). At a willingness‐to‐pay of €45,000 (≈US$50,000), the COFRAIL intervention was likely cost‐effective at approximately 46%.

### 3.4 Sensitivity Analysis

**FIGURE 3:**
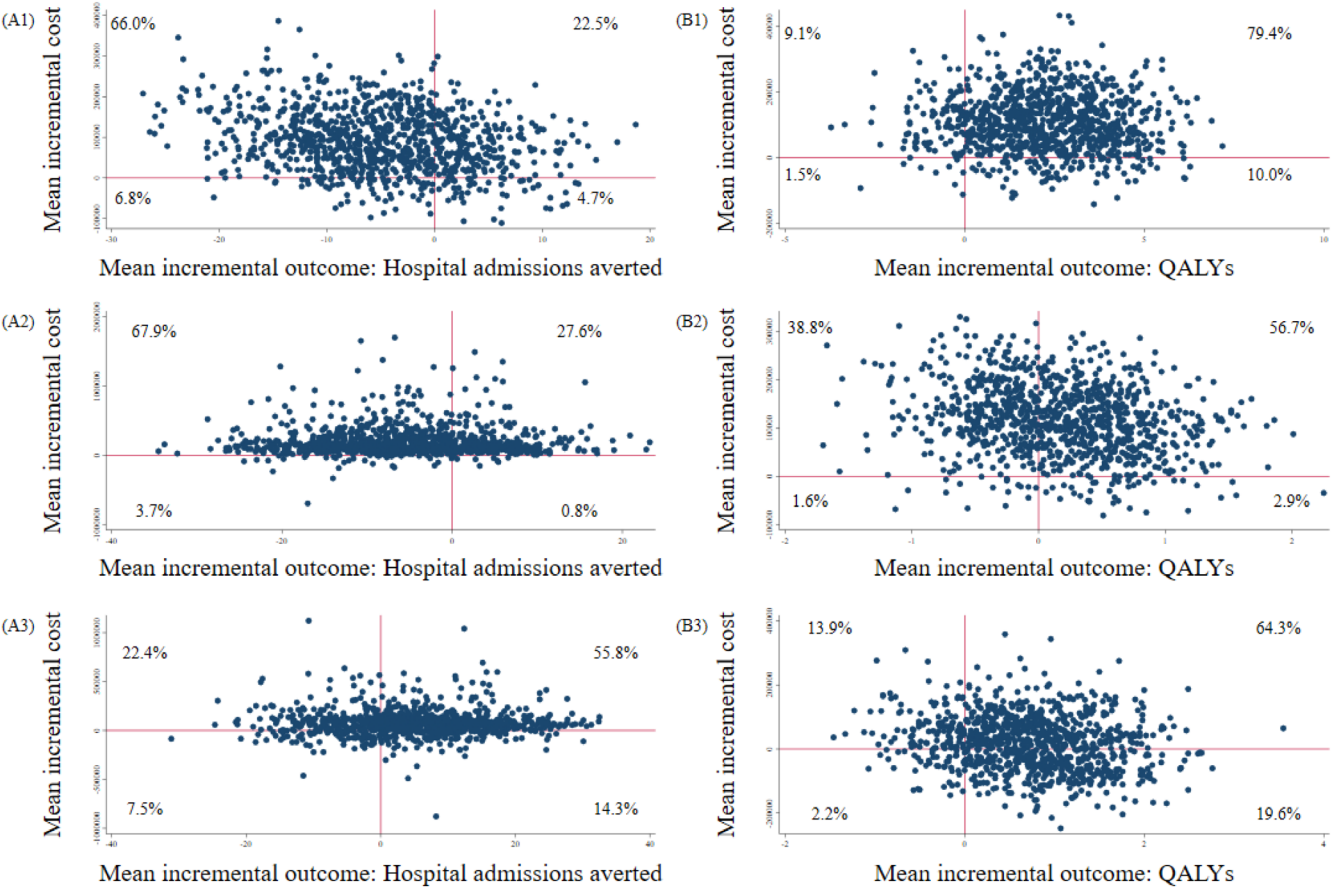
Cost‐effectiveness plane (A) and Cost‐utility plane (B) on 100 patients across sensitivity analyses. CEA, cost‐effectiveness analysis; CUA, cost‐utility analysis; ICER, incremental cost‐effectiveness ratio; ICUR, incremental cost‐utility ratio; QALYs, quality‐adjusted life years; SA, sensitivity analysis. In SA‐I (A1/B1), analysis was performed without adjustments; in SA‐II (A2/B2), analysis was performed with the per‐protocol population; in SA‐III (A3/B3), analysis was performed with complete information of the per‐protocol population (without multiple Imputation). In SA‐II (A2/B2), 385 of 521 patients were included (intervention group: 200 of 272, control group: 185 of 249); in SA‐III (A3/B3), 288 of 521 patients were included (intervention group: 150 of 272, control group: 138 of 249).

The results of sensitivity analyses SAI–SAIII are illustrated on cost‐effectiveness/cost‐utility planes in Figure 3, denoted as A1–A3 (cost‐effectiveness plane) and B1 to B3 (cost‐utility plane). Similar to the base case, the COFRAIL intervention was either dominated by usual care (A1, A2) or associated with more costs and higher QALYs than usual care (B1–B3).

Mean incremental cost‐outcome pairs appeared most frequently on the upper left quadrant of the cost‐effectiveness plane (66% in A1, 68% in A2, exception: 22% in A3) and on the upper right quadrant of the cost‐utility plane (79% in B1, 57% in B2, and 64% in B3). Although we observed the relative frequencies to decrease compared to the base case result, yet they remained relatively high towards relative frequencies in other quadrants. The results varied most in A3 as the COFRAIL intervention shifted towards a more costly but also more effective intervention in terms of hospital admissions averted than usual care (as opposed to the base case).

We reported the incremental cost‐effectiveness ratio of SA‐III (€23,489/hospital admission averted or US$27,550/hospital admission averted) and the incremental cost‐utility ratio of SA‐I to SA‐III (€51,491–€682,406/QALY or US$ 60,394–US$ 800.392/QALY) elsewhere (shown in Table S6). The results of additional analyses aligned with the majority of previous results, that is, the COFRAIL intervention was either dominated (SA‐IV) or associated with additional costs and additional QALYs (incremental cost‐utility ratio of SA‐V = €70,907/QALY or US$83,167) (shown in Table S6).

## 4 Discussion

This is the first cluster‐randomized controlled trial‐based health economic evaluation to assess the cost‐effectiveness of family conferences on deprescribing with joint prioritization of treatment goals conducted by general practitioners treating frail older care‐dependent patients with polypharmacy living at home.

At the 12‐month follow‐up, the COFRAIL intervention tended to be associated with higher costs but also (slightly) higher QALYs than usual care. This result was robust as it was replicated with bootstrapping in different sensitivity analyses with a frequency higher than 50% (57%–91%) on the plotted cost‐utility planes.

From descriptive analysis we know that healthcare costs (€11,464/US$13,446) rather than intervention costs (€391/US$459) were decisive in creating higher annual aggregated costs per capita in the intervention group compared to the control group (€10,722/US$12,576). The COFRAIL intervention did not seem to have a notable impact on healthcare resource use as higher healthcare costs were observed in the intervention group both at baseline and 12 months after baseline compared to the control group. The advantage of the intervention group toward the control group in terms of QALYs may be explained by the impact of the shared decision‐making process between general practitioners, patients, and caregivers (via family conferences) on health‐related quality of life. However, the slight differences in QALYs lead to high variable incremental cost‐utility ratio estimates across analyses.

Based on the highest commonly used willingness‐to‐pay threshold per QALY gained in the literature (€45,000/US$50,000 [35]), the probability of the COFRAIL intervention being cost‐effective with a base case incremental cost‐utility ratio of €50,966/QALY (US$59.778/QALY) was approximately 46%. To our knowledge, a cluster‐randomized controlled trial‐based health‐economic evaluation in Ireland [36] is the only intervention so far that also considered patients' preferences and came to a similar conclusion, finding the intervention to be more effective in terms of QALY gained but also more costly versus the comparator after 12 months. The study focused on deprescribing potentially inappropriate medications in older adults independently of frailty and polypharmacy. However, incremental cost‐utility ratio estimates were observed to range lower (incremental cost‐utility ratio≈€13,000–€37,000/QALY)/(incremental cost‐utility ratio≈US$15,250 to US$43,400/QALY) compared to our results, with a probability between 55% and 76% to be cost‐effective at a willingness‐to‐pay of €45,000 (≈US$50,000) per QALY gained. We explain the discrepancy by the health state of the COFRAIL population, as people with frailty and polypharmacy would require more additional costs to gain a QALY than the general older population.

Our health economic evaluation showed that the COFRAIL intervention was more likely to have a beneficial impact on costs associated with QALYs (cost‐utility analysis) than costs associated with hospital admissions as a measure for patient safety (cost‐effectiveness analysis). In the latter, we observed the COFRAIL intervention to be dominated by usual care (higher costs and no improvement in patent safety) as depicted in most cost‐effectiveness planes with a relative frequency varying between 28% and 78%. Regarding that our approach to analyzing hospital admissions in the base case differed from the COFRAIL study [10] with respect to defining the intention‐to‐treat population, choosing the data source, dealing with missing information, and constructing the regression model, no relevant differences appeared regarding this outcome.

So far, all health economic evaluations of interventions focusing on the pharmacotherapy under consideration of patients' preferences in older adults (not limited to living at home, frailty, and polypharmacy in some cases), either trial‐based [8, 9, 36] or model‐based [6, 7], had a study length of 6 months [8, 9] to 12 months [6, 7, 36]. Long‐term impacts beyond the follow‐up of 12 months still remain unknown. It is questionable whether QALYs in older adults with frailty and polypharmacy will increase, thus improving cost‐effectiveness in the long run considering the life expectancy of this population. A review in the field of palliative care discussed the use of QALYs in health economic evaluation towards the end of life [37]. Supporters believe that QALYs do matter and gains are possible even in populations with a low life expectancy and even if the intervention is not meant to extend life [38]. Therefore, investigating the long‐term impacts of the COFRAIL intervention on QALYs and on cost‐effectiveness could be the focus of future research. In addition, possible subgroups could be derived from the application of the geriatric toolbox [11], especially regarding different physical and mental limitations of frail older patients.

### 4.1 Strengths

A strength of this study was that we performed both a cost‐effectiveness analysis and cost‐utility analysis to understand the reach of the COFRAIL intervention on patient‐relevant outcomes and on costs associated with the intervention and healthcare resource use from the perspective of the German Social Insurance (i.e., statutory health and statutory long‐term care insurance). This perspective was chosen because the costs incurred for the intervention are to be borne by the social insurance and the target population is already retired, which means that there are no indirect costs due to productivity losses that would favor a societal perspective.

We addressed challenges in the dataset such as missing information and the nature of cost and outcome data (non‐normally distributed) by using multiple imputation techniques and by applying GLM regressions (based on distribution). We considered the cluster in the analysis by including the medical specialty of the family practice in GLM regressions. We assessed uncertainty in sensitivity analyses by plotting the results of the bootstrap procedure (simulated random resampling) on cost‐effectiveness plane and cost‐utility plane.

Furthermore, we investigated uncertainty surrounding our outcome parameters in additional analyses. As we calculated hospital admissions from a self‐assessment questionnaire in the base case, our outcome data might be prone to some degree of recall bias, which we believe was mitigated by using solely data from the questionnaire of the general practitioner in the sensitivity analysis. Calculating QALYs based on the EQ‐visual analogue scale enabled us to avoid rescaling of values, which was necessary in the presence of negative values when using data from the EQ‐5D‐5L questionnaire. We believe these issues to have no great impact on our data as the results of both additional analyses were similar to the base case results, that is, the COFRAIL intervention either being dominated or associated with additional costs per QALY gained compared to usual care. Since the health economic evaluation was performed alongside a cluster‐randomized controlled trial, outcome and cost data were both collected from the same population under trial‐based circumstances, which constitutes another strength.

### 4.2 Limitations

A limitation of our health economic evaluation was that dropouts (SA‐II) and missing information (SA‐III) seemed to be an issue in our dataset (in particular in the cost‐utility analysis) despite both groups being equally affected: the population reduced to 74% in both groups in SA‐II (intervention group: 200 of 272, control group: 185 of 249) and to around 55% in SA‐III (intervention group: 150 of 272, control group: 138 of 249). A higher drop‐out rate was observed among patients when study nurses changed data collection from home visits to telephone surveys during the COVID‐19 pandemic [10].

## 5 Conclusion

We conclude from this health economic evaluation that in older care‐dependent patients living at home with frailty and polypharmacy, family conferences on deprescribing with joint prioritization of treatment goals are more likely to affect QALYs than hospital admissions after 12 months of observation.

The COFRAIL intervention tended to be more costly but also more effective in terms of QALYs gained than usual care. Cost‐effectiveness was less likely observed at commonly used willingness‐to‐pay thresholds per QALY gained.

It remains unclear how cost‐effectiveness will change in the long run. Future research is necessary to determine whether the COFRAIL intervention is suitable in primary care.

## Author Contributions

Funding of the COFRAIL study was obtained by S. Wilm, P. Thürmann, B. Wiese, A. Altiner (Institute of General Practice, University Medical Center Rostock, Rostock, Germany), and G. Meyer (Institute for Health and Nursing Science, Medical Faculty, Martin Luther University Halle‐Wittenberg, Halle (Saale), Germany). To the conceptualization of the health economic evaluation of COFRAIL contributed J. Montalbo, C.‐M. Dintsios, and A. Icks. Data were provided by B. Wiese. Data related to pharmaceuticals were prepared by M. Gogolin (Department of Clinical Pharmacology, School of Medicine, Faculty of Health, Witten/Herdecke University, Witten, Germany). Data for the health economic evaluation were prepared by J. Montalbo. The analysis was performed by J Montalbo and discussed with C.‐M. Dintsios, and A. Icks. The manuscript was drafted by J. Montalbo and C‐M. Dintsios and revised by A. Icks, J. Abraham, E. Drewelow, M. Ritzke, B. Wiese, P. Thürmann, and collegues of the Department of Clinical Pharmacology, School of Medicine, Faculty of Health, Witten/Herdecke University, Witten, Germany (M. Gogolin, and V. Bencheva). The final paper was read and approved by all authors.

## Ethics Statement

Written informed consent was obtained from participants of the COFRAIL study. The COFRAIL study was reviewed and approved by the Ethics Committee at the Medical Faculty of the University of Rostock, approval number A2018‐0151, and by the Ethics Committee at the Medical Faculty of the Heinrich‐Heine‐University Düsseldorf, approval number 2018–283.

## Conflicts of Interest

The authors declare no conflicts of interest.

## Supporting information


**Supplementary Table S1.** Prices categorized by type of costs in 2019.
**Supplementary Table S2.** Our approach to quantify and evaluate healthcare resource use.
**Supplementary Table S3.** Imputation model for the base case.
**Supplementary Table S4.** Data preparation prior to multiple imputation.
**Supplementary Table S5.** Imputation model for sensitivity analyses.
**Supplementary Table S6.** Results of sensitivity analyses on 100 patients.
**Supplementary Figure S1.** Distribution of healthcare costs at baseline (A), 6 months after baseline (B), and 12 months after baseline (C).
Supplementary References.

## Data Availability

Supporting Information to this article can be found online. The data cannot be made available, owing to the European Union General Data Protection Regulation.
