# Supplementary material for: Cost‐Effectiveness of Family Conferences to Reduce Polypharmacy in Frail Older Adults
Source: J Am Geriatr Soc. 2025 Jun 27;73(10):3009–20. doi: 10.1111/jgs.19606 (PMC12554836; doi:10.1111/jgs.19606)
Supplement: Supplementary file 1 — Supplementary Table S1. Prices categorized by type of costs in 2019. Supplementary Table S2. Our approach to quantify and evaluate healthcare resource use. Supplementary Table S3. Imputation model for the base case. Supplementary Table S4. Data preparation prior to multiple imputation. Supplementary Table S5. Imputation model for sensitivity analyses. Supplementary Table S6. Results of sensitivity analyses on 100 patients. Supplementary Figure S1. Distribution of healthcare costs at baseline (A), 6 months after baseline (B), and 12 months after baseline (C). Supplementary References. [file JGS-73-3009-s001.pdf]

Supplemental Materials

Supplementary Table 1: Prices categorized by type of costs in 2019

**Cost items**	**Price per unit**	**Source**
Outpatient medical care		
Primary physicians	€67.47/treatment case	[1]
Internists	€147.14/ treatment case	
Gynecologists	€50.17/treatment case	
Urologists	€51.78/treatment case	
Orthopedists	€54.42/treatment case	
Surgeons	€75.67/treatment case	
Radiologists	€79.69/treatment case	
Otolaryngologists	€46.22/treatment case	
Oculists	€57.01/treatment case	
Dermatologists	€39.57/treatment case	
Neurologists	€67.55/treatment case	
Psychiatrists & psychotherapists	€307.98/treatment case	
Anesthesiologists	€163.71/treatment case	
Dentists	€105.64/treatment case	[2]
Therapeutic treatment		
Physiotherapists	€21.84/treatment unit	[3]
Occupational therapists	€47.42/treatment unit	
Speech therapists	€52.48/treatment unit	
Podologists	€33.92/treatment unit	
Hospital ambulances (emergency)	€12.99/treatment case	[4]
Rehabilitation		
Outpatient	€66.28/day	[5, 6]
Inpatient	€157.74/day	
Support in households		
Domestic help	€52.89/day	[7, 8]
Outpatient care	€52.89/day	
Hospital admissions	€3,927.28/treatment case - 10.00€/day	[9, 10]
Pharmaceuticals	varying	[10, 11]
Medical technical aids	€152.73/prescription	[12]
Services of the long-term care insurance		

Care aids	€40/month	[13]
Relief amount	PGI: €125/month	
Care allowance	PGI: -<br>PGII: €316/month<br>PGIII: €545/month<br>PGIV: €728/month<br>PGV: €901/month	
Outpatient care (benefit-in-kind)	PGI: -<br>PGII: €689/month<br>PGIII: €1,298/month<br>PGIV: €1,612/month<br>PGV: €1,995/month	
Inpatient care (short-term care)	PGI: -<br>PGII-PGV: €134.33/month	
Combination of care allowance and benefit-in-kind	50% of care allowance, 50% of benefit-in-kind	
Combination of care-allowance and short-term care	50% of care allowance, 100% of short-term care	
Intervention		
Time expenditure (general practitioners, scientific staff)	€59.06€/hour	[14, 15]
Time expenditure (staff at care centers, medical assistants)	€24.52€/hour	
Travelling expenses	€3.58/0-2km<br>€6.65/3-5km<br>€10.23/6-10km<br>€15.34/>10km	[16]
Materials/printing/postage, training (room and catering)	Actual costs	Study center

Abbreviation: Level of care dependency (PG)

Supplementary Table 2: Our approach to quantify and evaluate healthcare resource use

Cost items	Quantification	Evaluation
Outpatient medical care	Published sources from German statistics do not quantify the number of visits but the number of treatment cases, which is defined as the treatment of the same insured person by the same healthcare provider in a calendar quarter at the expense of the same health insurance company. As the number of visits was quantified in the COFRAIL study, we translated visits to treatment cases assuming that 1 visit equaled 1 treatment case per calendar quarter, whereas 2 visits equaled 2 treatment cases, and ≥3visits equaled 3 treatment cases.<br>We considered healthcare resource use only if frequency exceeded 1%.	Price for physician groups was calculated by dividing the fee turnover by the number of treatment cases [1].<br>For psychiatrists & psychotherapists (medical/psychological), we summed up the fee turnover of both groups as well as the number of treatment cases before calculating the ratio.<br>Price for dentists was calculated by dividing the billed amounts of dental services by the number of dental treatments [2].
Therapeutic treatments	We considered healthcare resource use only if frequency exceeded 1%.	Price was calculated by dividing the therapists' gross turnover by the therapists' treatment units [3]. Out-of-pocket payments were deducted from the price to receive the price from the SHI perspective.<br>To calculate out-of-pocket payments by therapist, total out-of-pocket payments were divided by the total treatment units across all therapists. Afterwards, the result was weighted with respect to the therapists' share of treatment units.
Hospital ambulances (emergency)	-	Price based on emergency fee (fee item 01210 of the "Einheitlicher Bewertungsmaßstab"(EBM)) [4].
Rehabilitation	-	Price calculated as described in Bock et al. [17] by dividing the outpatient (inpatient) expenditures [6] by the sum of outpatient (inpatient) days used for rehabilitation and prevention [5].
Support in households	We considered healthcare resource use only if frequency exceeded 1%. As a consequence, domestic help and outpatient care were considered as support in the household. We focused on	Price was calculated by dividing the sum of total expenditures related to support in household [7] by the total days used for support in households [8].

	patients without or with a low level of care dependency (level 1). Otherwise, support in the household would have been covered by the services of the long-term care insurance [18].	
Hospital admissions	-	Price based on the revenue volume per treatment case [9] excluding out-of-pocket payments of 10€ per each hospital day [10].
Pharmaceuticals	We narrowed healthcare resource due to medication intake down to medications with a pharmaceutical registration number (i.e., a nationwide standardized number to identify pharmaceuticals in Germany), to long-term medications, and to medications that were sold from January 1, 2017, onwards (considering the start of the COFRAIL study in 2018). We included medications with prescription assuming that they would be reimbursed by the statutory health insurance.	To evaluate medication intake, a ratio of corresponding pharmaceutical prices and a defined daily dose was calculated.<br>The pharmaceutical information was derived from the December 2017 database of the German Drug Index provided by the AOK Research Institute [11] and was matched with the patients' information via the pharmaceutical registration number, which was carried out by the Department of Clinical Pharmacology of the University in Witten/Herdecke.<br>Pharmaceutical prices were adjusted for out-of-pocket payments and pharmacy discounts (i.e., pharmacy price minus out-of-pocket payments of patients and minus pharmacy discount). Adjustments for discount of the drug manufacturer and discount specific to health insurance companies was not possible because this information is varying and kept confidential.<br>Out-of-pocket payments of patients included the positive differential between pharmacy price and the fixed amount paid by SHIs for a pharmaceutical. In addition, patients either<br>a) fully pay for pharmaceuticals if the pharmacy price is below €5,<br>b) or they have copayments of €5 if pharmacy price ranges between €5-50,<br>c) or copayments of 10% of the pharmacy price if it ranges between €51-100,<br>d) or copayments of €10 if pharmacy price is higher than €100 [10].

Medical technical aids	If patients claimed medical technical aids, we assumed that they received the benefit just once per time of data collection as quantities were not stated in the questionnaire.	Price was calculated by dividing medical technical aids expenditures by the number of prescriptions for medical technical aids [12].<br>Both components needed to be calculated for 2019 prior to division. We calculated the mean change in percentage from 2016 to 2018, multiplied it by the value given for 2018, and summed up the product with value given for 2018.
Services of the long-term care insurance	If patients claimed care aids, we assumed that they received the benefit just once per time of data collection as quantities were not stated in the questionnaire.	Prices based on the availability of care dependency of patients measured at baseline.
	If patients claimed services of the long-term care insurance (other than care aids), we assumed that they received the benefits constantly over time.<br>For patients with a low level of care dependency (level 1), we assumed that they would receive a benefit that corresponds to a relief amount [cf.13].<br>Patients with a higher level of care dependency (level 2 to 5) were eligible for care allowance, outpatient and inpatient care. Here, outpatient care was treated as benefit-in-kind whereas inpatient care was treated as short-term care.<br>Patients were also eligible to receive combined services (e.g., care allowance and benefit-in-kind, care allowance and short-term care) [13]. If patients stated combined services of short-term-care and benefit-in-kind, we only considered the latter.	Prices based on the level of care dependency of patients measured at baseline.
Time expenditure		We assumed general practitioners and scientific staff to be full-time employees working in leading positions (corresponding to group 1), and care center staff and medical assistants to

		be full-time employees working in positions as professionals (corresponding to group 3) in the healthcare sector [14].<br>We added social security contributions of the employer (9.30% statutory pension insurance; 1.25% statutory unemployment insurance; 7.30% SHI; 1.525% statutory care insurance) to gross hourly wages [15].

Supplementary Table 3: Imputation model for the base case

Missing data occurred in additional variables (at baseline), hospital admissions, utility weights, and costs (all at baseline, 6 months after baseline, and 12 months after baseline).

We applied multiple imputation by chained equations (MICE) [19, 20] as it allowed to deal with different missing data patterns [cf.21].

To keep the number of variables in the imputation model low as suggested by Hardt et al. [22], we included in the imputation model only variables that were also used in the analysis, i.e., costs (at baseline, 6 months after baseline, and 12 months after baseline, except for intervention costs), utility weights (at baseline, 6 months after baseline, and 12 months after baseline), hospital admissions (12 months after baseline), and additional variables (excluding those accounting for the general practitioner practice).

We imputed costs rather than quantities to be able to aggregate cost components and thus reduce the number of variables entering the imputation model. The imputation of cost components was less successful if the number of observations with positive costs was small. By aggregating cost components, we were able to avoid this problem. This required the dataset to have similar missing data patterns across cost components (i.e., having either complete information or no information on costs for each participant) and similar recall periods. We summed up costs related to hospital ambulances (emergency) with the costs related to rehabilitation.

If cost variables had a high number of zero observations, we applied a two-step imputation [23] by imputing, first, the probability of cost variables having positive values and, second, the cost variable itself conditioned on positive values. This approach was also used in regard to hospital admissions and utility weights. All cost variables entered the two-step imputation with one condition, except for hospital costs. Hospital costs was imputed in three steps: first, the probability of hospital admissions having positive values, second, the number of hospital admissions conditioned on positive values, and, third, hospital costs conditioned on the first and second step. No two-step imputation was necessary for outpatient medical care (at baseline, 6 months after baseline, and 12 months after baseline) and for pharmaceutical costs (at baseline). We imputed log-transformed costs in all imputation models as imputed values were more likely to be stable regardless of the amount of missing data [23]. However, log-transformed costs were transformed back for cost-effectiveness /cost-utility analysis.

We applied Predictive Mean Matching (pmm) procedures that randomly selected one of three of nearest observed values to replace missing values in costs, utility weights, and additional continuous variables (BMI). We also used a logit model to replace missing values in additional binary variables and in variables to estimate the probability within the two-step imputation.

The same seed of random number generator was specified for each imputation (i.e., 123). The imputation was performed separately according to treatment assignment to take into account possible differences [24]. Variables with missing values were replaced in the order starting from the lowest to the highest percentage of missing values. If the percentage of missing values was equal, additional variables were imputed first prior to utility weights, hospital admissions, and costs.

Additional variables with complete information prior to the imputation process were part of the prediction equation to replace missing information in variables. The prediction equation included additional variables to describe population characteristics such as sex, age, housing situation, and recruitment site (excluded: medical specialty and identification number of the general practitioners practice). After each imputation, the imputed variable was eligible to predict another variable with missing values and thus was also included in the prediction equation.

However, imputed costs were only used as additional predictor to replace missing information of respective costs measured in later time periods. For example, imputed costs related to outpatient medical care at baseline would be eligible as additional predictor to replace missing information in

outpatient medical care 6 months after baseline. Then, the imputed values at baseline and 6 months after baseline would both be eligible as predictor to replace missing information in outpatient medical care 12 months after baseline. Imputed values of outpatient medical care would not be eligible as predictor to replace missing values in other cost components, e.g., pharmaceutical costs. We followed this approach similarly when imputing utility weights and hospital admissions.

In some cases, predictor variables needed to be excluded from the prediction equation to achieve a successful imputation. Those variables were identified either by the statistical program or manually. The latter involved an assessment regarding whether the predictor was likely to be more closely associated with the variable being imputed than other predictor variables.

Supplementary Table 4: Data preparation prior to multiple imputation

Before using multiple imputation, we applied different approaches to deal with missing information depending on case:

Case	Approach	Applied to data
Patient stated "don't know/no answer" in reply to the question whether a healthcare resource use took place.	We assumed that healthcare resource was not claimed.	• Outpatient medical care<br>• Therapeutic treatment<br>• Hospital ambulance (emergency)<br>• Rehabilitation<br>• Support in the household<br>• Hospital admissions<br>• Medical technical aids and care aids<br>• Services of the long-term care insurance
Patient stated to have claimed healthcare resource use but stated "don't know/no answer" in reply to the question how often healthcare resource use took place or how long healthcare resource use was claimed.	We assumed the amount of healthcare resource use to equal the median value (with respect to the time of data collection).	• Outpatient medical care<br>• Therapeutic treatment<br>• Hospital ambulance (emergency)<br>• Rehabilitation<br>• Support in the household<br>• Hospital admissions, length of hospital stay<br>• Duration of medical intake
Patient stated to have claimed healthcare resource use but information was missing in reply to the question how often healthcare resource use took place or how long healthcare resource use was claimed (“don't know/no answer" was not stated)	We assumed the amount of healthcare resource use to equal the median value (with respect to the time of data collection).	• Support in the household<br>• Length of hospital stay

Supplementary Table 5: Imputation model for sensitivity analyses

Depending on the sensitivity analysis, we decided either to use the same imputed dataset as in the base case (applied to SA-I without adjustments), rerun multiple imputation with the same imputation model (applied to SA-IV with hospital admissions based on data of the general practitioner), or rerun multiple imputation with changes in the imputation model.

For the latter, we describe the changes of the imputation model from the base case:

Sensitivity analysis	Changes from the base case approach
Analysis based on the per-protocol-population (SA-II)	• the number of variables being imputed was smaller due to two reasons: first, more variables had complete information prior to multiple imputation, and second, no two-step imputation was necessary for pharmaceutical costs and utility weights (at baseline, 6 months after baseline, 12 months after baseline)<br>• in some cases, further predictor variables needed to be excluded from the prediction equation to achieve a successful imputation
Analysis with QALYs based on the EQ-visual analogue scale (SA-V)	• the number of variables being imputed was smaller because no two-step imputation was necessary for values of the EQ-visual analogue scale (at baseline, 6 months after baseline, 12 months after baseline)

In order to increase the comparability of SA-II and SA-V with the base case approach, we kept the order of variables with missing values being replaced starting from the lowest to the highest percentage similar to the order used in the base case approach. Furthermore, we generated the same number of imputed datasets as in the base case though the maximum percentage of missing values varied across sensitivity analyses.

Supplementary Table 6: Results of sensitivity analyses on 100 patients[a]

Analysis	CEA			CUA		
	Incremental cost	Incremental outcome	ICER	Incremental cost	Incremental outcome	ICUR
	€	Hospital admissions averted	€/Hospital admissions averted	€	QALYs	€/QALY
SA-I	94,615 [-59,092; 248,321]	-5.59 [-26.44; 15.25]	Dominated	113,281 [-67,226; 293,788]	2.20 [-1.62; 6.02]	51,491.36
SA-II[b]	319,704 [-948,196; 1,587,604]	-4.48 [-22.68; 13.71]	Dominated	109,185 [-35,251; 253,621]	0.16 [-1.09; 1.41]	682,406.25
SA-III[c]	114,625 [-486,722; 715,972]	4.88 [-15.04; 24.80]	23,488.73	17,357 [-136,313; 171,027]	0.81 [-0.52; 2.14]	21,428.4
SA-IV	109,540 [-332,35; 252,315]	-1.68 [-19.65; 16.29]	Dominated			
SA-V				112,742 [-23,525; 249,008]	1.59 [-1.81; 4.99]	70,906.92

Abbreviation: cost-effectiveness analysis (CEA), cost-utility analysis (CUA), incremental cost-effectiveness ratio (ICER), incremental cost-utility ratio (ICUR), sensitivity analysis (SA)

[a]In SA-I, analysis was performed without adjustments; in SA-II, analysis was performed with the per-protocol population; in SA-III, analysis was performed with complete data of the per-protocol population (without multiple Imputation); in SA-IV, the questionnaire of general practitioners was used to calculate hospital admissions; in SA-V, the values of the EQ-visual analogue scale was used to calculate QALYs

[b]385 of 521 patients were included (intervention group: 200 of 272, control group: 185 of 249)

[c]288 of 521 patients were included (intervention group: 150 of 272, control group: 138 of 249)

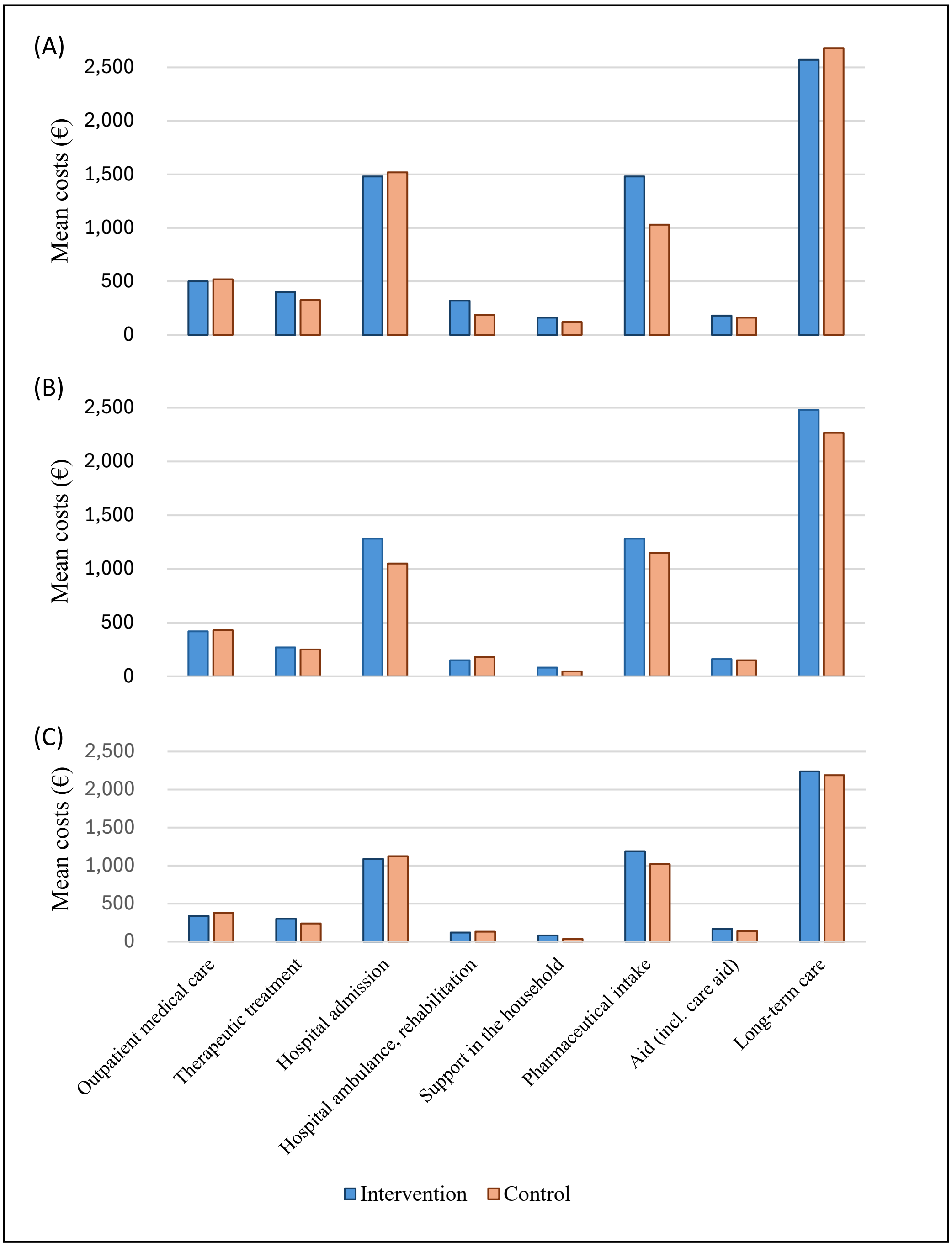

Supplementary Figure 1: Distribution of healthcare costs at baseline (A), 6 months after baseline (B), and 12 months after baseline (C)

References

1 Kassenärztliche Bundesvereinigung (National Association of Statutory Health Insurance Physicians) (KBV). Abrechnungsstatistik der Kassenärztlichen Bundesvereinigung (Billing statistics of the KBV): Honorarbericht nach §87c SGB V 1. Quartal 2013 bis 4. Quartal 2019 (Fee report according to §87c SGB V 1st quarter 2013 to 4th quarter 2019); 2021. Available from: URL: https://www.kbv.de/html/honorarbericht.php.

2 Kassenzahnärztliche Bundesvereinigung (National Association of Statutory Health Insurance Dentists) (KZBV). Jahrbuch 2020 (Yearbook 2020): Statistische Basisdaten zur vertragszahnärztlichen Versorgung (Basic statistical data on contractual dental care); 2020. Available from: URL: https://www.kzbv.de/kzbv-jahrbuch-2020-2.media.d31ec54df2c2e2592d7d45681edcfc0f.pdf.

3 GKV-Heilmittel-Informations-System (Statutory Health Insurance Medication Information System) (GKV-HIS). GKV-Heilmittel-Schnellinformation für Deutschland nach §84 Abs. 5 i.V. m. Abs. 8 SGB V (Information Overview for Germany according to §84 SGB V): Januar bis Dezember 2019 (January to December 2019); 2020. Available from: URL: https://www.gkv-heilmittel.de/fuer_vertragsaerzte/his_berichte/his_berichte.jsp?area=13&criterion1=3286304&criterion2=33.

4 Kassenärztliche Bundesvereinigung (National Association of Statutory Health Insurance Physicians) (KBV). Einheitlicher Bewertungsmaßstab (EBM) (Standardized Evaluation): Stand: 4. Quartal 2019 (4th quarter 2019); 2019. Available from: URL: https://www.kbv.de/html/arztgruppen_ebm.php.

5 Bundesgesundheitsministerium (Federal Ministry of Health) (BMG). KG 5 Statistik (KG 5 Statistics): Ergebnisse der Statistik KG 5, Vorsorge- und Rehabilitationsmaßnahmen 2019 der Gesetzlichen Krankenversicherungen (Results of the KG 5 Statistics, Preventive and Rehabilitation Measures 2019 of the Statutory Health Insurance); 2020. Available from: URL: https://www.bundesgesundheitsministerium.de/themen/krankenversicherung/zahlen-und-fakten-zur-krankenversicherung/geschaeftsergebnisse.html.

6 Bundesgesundheitsministerium (Federal Ministry of Health) (BMG). KJ 1 Statistik (KJ 1 Statistics): Gesetzliche Krankenversicherung- Endgültige Rechnungsergebnisse 2019 (Statutory Health Insurance- Final Calculations 2019); 2020. Available from: URL: https://www.bundesgesundheitsministerium.de/themen/krankenversicherung/zahlen-und-fakten-zur-krankenversicherung/finanzergebnisse.html.

7 Bundesgesundheitsministerium (Federal Ministry of Health) (BMG). Gesetzliche Krankenversicherung-Vorläufige Rechnungsergebnisse (Statutory Health Insurance-Preliminary Calculations): 1. bis 4. Quartal 2019 (1st to 4th quarter 2019); 2020 [cited 2021 December 21]

Available from: URL:
https://www.bundesgesundheitsministerium.de/themen/krankenversicherung/zahlen-und-fakten-zur-krankenversicherung/finanzergebnisse.html.

8 Bundesgesundheitsministerium (Federal Ministry of Health) (BMG). KG 2-Statistik (KG 2 Statistics): Leistungsfälle, Leistungszeiten und Leistungstage je -fall bei Haushaltshilfen der Versicherten in der gesetzlichen Krankenversicherung (Service Cases, Service Times, and Service Days per Case for Household Aids of Persons Insured in the Statutory Health Insurance system); 2021 [cited 2021 December 21] Available from: URL: https://www.gbe-bund.de/gbe/pkg_isgbe5.prc_menu_olap?p_uid=gast&p_aid=18409964&p_sprache=D&p_help=3&p_indnr=301&p_indsp=&p_ityp=H&p_fid=.

9 Statistisches Bundesamt (Federal Statistical Office) (StBA). H 1-Gesundheit (H 1-Health): DRG-Statistik 2010-2019- Vollstationäre Patientinnen und Patienten in Krankenhäusern (DRG Statistics 2010-2019: Full Inpatients in Hospitals); 2021.

10 Gesetz zur Modernisierung der gesetzlichen Krankenversicherung (GKV-Modernisierungsgesetz-GMG) (Statutory Health Insurance Modernisation Law); 2003. Available from: URL: https://www.bgbl.de/xaver/bgbl/start.xav?startbk=Bundesanzeiger_BGBl&start=//*%255B@attr_id=%2527bgbl103s2190.pdf%2527%255D#__bgbl__%2F%2F*%5B%40attr_id%3D%27bgbl103s2190.pdf%27%5D__1582107643968.

11 Wissenschaftliches Institut der AOK (AOK Research Institute) (WIdO). GKV-Arzneimittelindex (Drug index of the Statutory Health Insurance): Stammdatei plus (Master File Plus); 2020.

12 Buksch S, Osterkamp N, Wittkop C, Hoffmann N. Heil- und Hilfsmittelreport 2019 (Medicine and Healthcare products report 2019). In: Schriftenreihe zur Gesundheitsanalyse.

13 Bundesgesundheitsministerium (Federal Ministry of Health) (BMG); E-Mail; 2021 Oct 8.

14 Statistisches Bundesamt (Federal Statistical Office) (StBA). Verdienste und Arbeitskosten (Earnings and Labor Costs): Arbeitnehmerverdienste- 2019 (Employee Earnings 2019); 2020. Available from: URL: https://www.destatis.de/DE/Themen/Arbeit/Verdienste/Verdienste-Verdienstunterschiede/Publikationen/Downloads-Verdienste-und-Verdienstunterschiede/arbeitnehmerverdienste-jahr-2160230197004.html.

15 Deutsche Rentenversicherung Bund (German Statutory Pension Insurance) (DRV Bund). Rentenversicherung in Zeitreihen (Pension Insurance in Time Series): Oktober 2019 (October 2019); 2019. Available from: URL: https://www.deutsche-rentenversicherung.de/SharedDocs/Downloads/DE/Statistiken-und-Berichte/statistikpublikationen/rv_in_zeitreihen.pdf?__blob=publicationFile&v=3.

16 Verband der Privaten Krankenversicherung (Association of Private Health Insurers) (PKV-Verband). Gebührenordnung für Ärzte (Scale of fees for physicians) (GOÄ): mit verkürzten

Leistungsbezeichnungen (with abbreviated service descriptions); 2013 [cited 2021 December 21] Available from: URL: https://www.derprivatpatient.de/sites/default/files/gebuehrenordnung-fuer-aerzte.pdf.

17 Bock J-O, Brettschneider C, Seidl H, Bowles D, Holle R, Greiner W*, et al.* Ermittlung standardisierter Bewertungssätze aus gesellschaftlicher Perspektive für die gesundheitsökonomische Evaluation: Calculation of standardised unit costs from a societal perspective for health economic evaluation. Das Gesundheitswesen : Sozialmedizin, Gesundheits-System-Forschung, medizinischer Dienst, public health, öffentlicher Gesundheitsdienst, Versorgungsforschung 2015; 77(1): 53–61.

18 Sozialgesetzbuch (SGB V) Fünftes Buch Gesetzliche Krankenversicherung- § 38 SGB V Haushaltshilfe (Social Code (SGB V), Book 5: Statutory Health Insurance- § 38 SGB V Household Support); 1989 [cited 2021 December 20] Available from: URL: https://www.sozialgesetzbuch-sgb.de/sgbv/38.html.

19 Raghunathan TW, Lepkowksi JM, Van Hoewyk J., Solenbeger P. A multivariate technique for multiply imputing missing values using a sequence of regression models. Survey Methodology 2001; 27: 85–95.

20 van Buuren S. Multiple imputation of discrete and continuous data by fully conditional specification. Statistical Methods in Medical Research 2007; 16(3): 219–42.

21 Bouhlila DS, Sellaouti F. Multiple imputation using chained equations for missing data in TIMSS: a case study. Large-scale assessments in education 2013; 1(1): 1–33.

22 Hardt J, Herke M, Leonhart R. Auxiliary variables in multiple imputation in regression with missing X: A warning against including too many in small sample research; 2012. Available from: URL: http://bmcmedresmethodol.biomedcentral.com/articles/10.1186/1471-2288-12-184.

23 MacNeil Vroomen J, Eekhout I, Dijkgraaf MG, van Hout H, Rooij SEd, Heymans MW*, et al.* Multiple imputation strategies for zero-inflated cost data in economic evaluations: Which method works best? The European journal of health economics : HEPAC : health economics in prevention and care 2016; 17(8): 939–50.

24 Royston P. Multiple imputation of missing values: further update of ice, with an emphasis on categorical variables. Stata J 2009; 9(3): 466.
